# Patterns of hybridization among cutthroat trout and rainbow trout in northern Rocky Mountain streams

**DOI:** 10.1002/ece3.1887

**Published:** 2016-01-11

**Authors:** Kevin S. McKelvey, Michael K. Young, Taylor M. Wilcox, Daniel M. Bingham, Kristine L. Pilgrim, Michael K. Schwartz

**Affiliations:** ^1^ USDA Forest ServiceRocky Mountain Research StationNational Genomics Center for Wildlife and Fish Conservation800 East Beckwith AvenueMissoulaMontana59801; ^2^ Division of Biological SciencesUniversity of MontanaMissoulaMontana59812; ^3^ Rogue Biological Consultants215 NW 22nd Pl Suite 207PortlandOR97217

**Keywords:** cutthroat, hybridization, *Oncorhynchus*, SNP, swarm

## Abstract

Introgressive hybridization between native and introduced species is a growing conservation concern. For native cutthroat trout and introduced rainbow trout in western North America, this process is thought to lead to the formation of hybrid swarms and the loss of monophyletic evolutionary lineages. Previous studies of this phenomenon, however, indicated that hybrid swarms were rare except when native and introduced forms of cutthroat trout co‐occurred. We used a panel of 86 diagnostic, single nucleotide polymorphisms to evaluate the genetic composition of 3865 fish captured in 188 locations on 129 streams distributed across western Montana and northern Idaho. Although introgression was common and only 37% of the sites were occupied solely by parental westslope cutthroat trout, levels of hybridization were generally low. Of the 188 sites sampled, 73% contained ≤5% rainbow trout alleles and 58% had ≤1% rainbow trout alleles. Overall, 72% of specimens were nonadmixed westslope cutthroat trout, and an additional 3.5% were nonadmixed rainbow trout. Samples from seven sites met our criteria for hybrid swarms, that is, an absence of nonadmixed individuals and a random distribution of alleles within the sample; most (6/7) were associated with introgression by Yellowstone cutthroat trout. In streams with multiple sites, upstream locations exhibited less introgression than downstream locations. We conclude that although the widespread introduction of nonnative trout within the historical range of westslope cutthroat trout has increased the incidence of introgression, sites containing nonadmixed populations of this taxon are common and broadly distributed.

## Introduction

Introductions of nonnative species are of global conservation concern (Clavero and García‐Berthou 2005). Although much of the focus has been directed at predation or competition resulting from nonnative species invasions, hybridization with nonnative taxa has been increasingly emphasized (Perry et al. 2002; Seehausen 2006). Hybridization is nonintrogressive if hybrid offspring fail to develop or suffer an extreme fitness penalty such as infertility, and represents wasted reproductive effort for individuals of the native species (Rhymer and Simberloff 1996). Alternatively, introgressive hybridization results, if hybrid offspring survive, are fertile, and contribute their alleles to future generations. Although introgressive hybridization can lead to a number of outcomes, such as the creation of new species (e.g., Nolte and Tautz 2010), the development of hybrid zones is more common. These hybrid zones often take the form of tension zones composed of parental forms and less‐fit hybrids, or of zones of bounded hybrid superiority in which hybrids are locally more fit than either parent. (Mallet 2005). A third possibility, particularly prominent in the conservation literature, involves the development of globally superior (Culumber et al. 2012; Arnold 2015) or equally fit hybrids that generate hybrid swarms (Mayr 1963). In this case, nonnative genes freely mix and eventually lead to a population that consists solely of introgressed individuals in which the genes of both species are distributed throughout all members of the population.

Fishes are regarded as particularly prone to introgressive hybridization – even between highly divergent taxa (Bossu and Near 2013; Montanari et al. 2014) – because of their reliance on external fertilization and the absence of strong prezygotic reproductive barriers (Hubbs 1955; Campton 1987; Scribner et al. 2001). Because introductions of nonnative fishes are an issue worldwide (Rahel 2007), their hybridization with native species is a mounting concern (Aboim et al. 2010). This is particularly true for fishes in the family Salmonidae that are among the most widely introduced species for sport fishing and aquaculture (Crawford and Muir 2008; Gozlan et al. 2010). For example, Allendorf and Leary (1988) concluded that the greatest threat to indigenous populations of the various subspecies of cutthroat trout (*Oncorhynchus clarkii*) in the western United States was the introduction of nonindigenous forms of that species or, more commonly, the congeneric rainbow trout (*O. mykiss*). Following such introductions, observations of introgressed individuals are the norm (Rubidge et al. 2001; Metcalf et al. 2008), with most populations of taxa such as westslope cutthroat trout (*O. c. lewisi*) thought to be hybridized with nonnative rainbow trout or Yellowstone cutthroat trout (*O. c. bouvieri*; Allendorf et al. 2001, 2013; Shepard et al. 2005; Muhlfeld et al. 2009b). Because matings with and among hybrid fish beget more hybrids (Epifanio and Philipp 2001), a process labeled the “ratchet effect” (Allendorf et al. 2004), introgression between native cutthroat trout and nonnative taxa has been regarded as irreversible (Leary et al. 1984) and the development of hybrid swarms to be common and inevitable (Leary et al. 1995; Allendorf et al. 2004; Ostberg and Rodriguez 2006). This logic implies that streams containing admixed individuals at any location will eventually have admixed individuals throughout a population (Boyer et al. 2008).

There are, however, indications that the situation may be more complex and that some populations of westslope cutthroat trout may be resistant to hybridization. For example, Muhlfeld et al. (2014) found that within the North Fork Flathead River, a basin where an estimated 20 million rainbow trout had been introduced over a period of at least 70 years, 70% of the sites sampled had less than 1% introgression, and none of the sites from the upper ~50% of the basin showed signs of rainbow trout introgression (Muhlfeld et al. 2014; Table S1). Similarly, in tributaries of the Middle Fork Clearwater River basin in northern Idaho, Weigel et al. (2003) found nonadmixed populations of westslope cutthroat trout at 36% of the sites despite extensive stocking of rainbow trout in the preceding half‐century. In a broad survey covering the range of westslope cutthroat trout, Shepard et al. (2005) found that 58% of tested samples showed no evidence of hybridization, a pattern even more evident in the smallest streams (Shepard et al. 2005, table 5).

Geography is thought to influence the outcome of introgressive hybridization between cutthroat trout and rainbow trout. Some basins where these species are naturally sympatric, for example, the Salmon and Clearwater River basins in Idaho and several coastal river basins in Oregon and Washington, are regarded as population strongholds for cutthroat trout (Johnson et al. 1999; Shepard et al. 2005). It has been argued that where the historical ranges of these species overlap, hybridization is inconsequential because of strong pre‐ and postzygotic isolating mechanisms (Behnke 1992; Allendorf et al. 2004, 2005), such as differences in the timing of spawning and divergent life histories that might select against hybrids (Taylor 2004). Hybridization between these species in areas of natural sympatry, however, has been regularly observed in both pristine habitats that have rarely or never been stocked with rainbow trout (Wenberg and Bentzen 2001; Young et al. 2001; Ostberg et al. 2004; Kozfkay et al. 2007; Williams et al. 2007) as well as those with frequent stocking (Docker et al. 2003; Weigel et al. 2003; Bettles et al. 2005; Heath et al. 2010; Loxterman et al. 2014). In addition, there are often upstream–downstream differences in the prevalence of introgression, regardless of whether rainbow trout are indigenous to a basin (Ostberg et al. 2004; Gunnell et al. 2008). Nonetheless, because rainbow trout stocking has been widespread and intensive where both species are native (Idaho stocking data are available at http://fishandgame.idaho.gov/public/fish/stocking/; Montana stocking data are available at http://fwp.mt.gov/fishing/mFish/), high levels of introgression and the formation of hybrid swarms might be typical where these species co‐occur, regardless of their origins or location within the watershed.

Patterns of introgression are further complicated by the presence of Yellowstone cutthroat trout, which have also been widely introduced within the historical range of westslope cutthroat trout (Gresswell and Varley 1988). Unlike rainbow trout and cutthroat trout, no subspecies of cutthroat trout naturally co‐occur, and they have similar habitat preferences and spawning behavior (Behnke 1992). In addition, most subspecies of cutthroat trout have diverged relatively recently (1–2 million years; Loxterman and Keeley 2012), and thus, reproductive barriers have had little time to develop. Consequently, introductions of one form of cutthroat trout into the range of another might be expected to lead to a high incidence of introgressive hybridization and potentially of hybrid swarms (Leary et al. 1995).

Regardless of the taxa involved, detecting and characterizing the patterns of introgression is contingent on using a sufficient number of genetic markers to precisely diagnose the hybrid status of individual fish – particularly when levels of introgression are low – and on spatially distributed sampling within and among streams (Anderson et al. 2008). Because relatively few genetic markers have been employed in most analyses of cutthroat trout and rainbow trout introgression (Table 1), the distribution of alleles of both parental forms among individuals in admixed populations is uncertain (Boecklen and Howard 1997). Nonrandom allelic distributions, such as the presence of nonintrogressed parental forms in admixed populations, can indicate that introgression is recent or occasional, or might imply resistance to introgression arising from assortative mating, reduced hybrid fitness, or parental fish dispersing from elsewhere in a watershed (Jiggins and Mallet 2000). Larger numbers of genetic markers also permit precise descriptions of individual genotypes instead of the more generic conclusions about introgression based on pooled samples (e.g., the hybrid index; Campton and Utter 1985). Similarly, multiple samples from a single stream and among many streams can shed light on the variation in admixture within populations (Culumber et al. 2011) and permit a better understanding of the structure of hybrid zones across a species range (Barton and Hewitt 1985).

**Table 1:** Sources of data on introgression between native and introduced cutthroat trout and rainbow trout in western North America for which the presence of parental forms could be determined. Species in bold were introduced. Markers are the number of nuclear loci used to estimate introgression. Introgression at sites was classified as present or absent based on criteria used by the original authors (the number of sites lacking parental forms is in parentheses)

Species^a^	Source	Markers	Introgression	
			Present	Absent
**RT** × PCT	Busack and Gall (1981)	10	2 (0)	0
RT × CCT	Campton and Utter (1985)	4	8 (1)	0
**YCT** × WCT	Gyllensten et al. (1985)	11	2 (2)	0
**YCT** × **WCT**	Marnell et al. (1987)^b^	6	3 (1)	0
**RT** × LCT	Bartley and Gall (1991)	5	1 (0)	3
**YCT** × WCT	Forbes and Allendorf (1991)	12	3 (3)	0
RT × CCT	Young et al. (2001)	23	5 (0)	11^c^
RT × CCT	Docker et al. (2003)	4	6 (0)	4
RT × CCT	Ostberg et al. (2004)	22	7 (0)	0
**RT** × LCT	Peacock and Kirchoff (2004)	10	3 (1)	1
**RT** × WCT	Rubidge and Taylor (2004)	4	18 (0)	5
RT × CCT	Baumsteiger et al. (2005)	7	3 (0)	0
**RT** × WCT	Ostberg and Rodriguez (2006)	4	14 (0)	4^c^
RT × WCT	Kozfkay et al. (2007)	3	14 (0)	3
RT × CCT	Williams et al. (2007)	4	8 (0)	5^c^
**RT** × WCT	Boyer et al. (2008)	7	17 (1)	14
**RT** × YCT	Gunnell et al. (2008)	7	16 (0)	12
**RT** × CRCT	Metcalf et al. (2008)	7	2 (0)	2
**RT** × WCT	Bennett and Kershner (2009)^d^	4	11 (0)	3
**RT** × WCT	Muhlfeld et al. (2009a)	16	1 (0)	0
RT × CCT	Heath et al. (2010)^e^	7	29 (0)	6
**RT** × WCT	Rasmussen et al. (2010)	3	16 (0)	7^c^
**RT** × YCT	Kovach et al. (2011)	14	10 (0)	0
**WCT** × RT	Neville and Dunham (2011)	7	14 (0)	27^c^
**RT** × WCT	Ostberg and Chase (2012)^f^	4	6 (0)	2^c^
RT × CCT	Buehrens et al. (2013)	4	1 (0)	0
RT × WCT	Loxterman et al. (2014)	6	25 (0)	7
**RT** × WCT	Kovach et al. (2015)^g^	8	2 (0)	0
**RT** × **YCT** × LCT	Pritchard et al. (2015)	46	7 (4)	26

a Species abbreviations: CCT, coastal cutthroat trout; CRCT, Colorado River cutthroat trout *O. c. pleuriticus*; LCT, Lahontan cutthroat trout *O. c. henshawi*; PCT, Paiute cutthroat trout *O. c. seleneris*; RT, rainbow trout; WCT, westslope cutthroat trout; YCT, Yellowstone cutthroat trout.

b WCT and YCT were both introduced to two of three lakes; WCT were native to the third.

c One or more sites had only nonintrogressed rainbow trout.

d Seven sites also used by Rubidge and Taylor (2004).

e Five sites also used by Docker et al. (2003), but sampled in a different year.

f Six sites also used in Ostberg and Rodriguez (2006).

g One site also used in Muhlfeld et al. (2009a).

We assessed patterns of introgression, with an emphasis on ascertaining the prevalence of hybrid swarms, between rainbow trout and cutthroat trout and between native and introduced cutthroat trout in western North America. First, we conducted a literature review to evaluate the observed frequency of hybrid swarms in contact zones between these taxa throughout this region. Second, we used a panel of 86 species‐diagnostic, single nucleotide polymorphisms (SNPs) to characterize levels of admixture of individual fish and patterns of introgression between native westslope cutthroat trout, introduced Yellowstone cutthroat trout, and native or introduced rainbow trout in western Montana and northern Idaho. To evaluate spatial patterns, we also compared levels of admixture between sites within individual streams, and between streams inside and outside the historical range of rainbow trout. Additionally, we compared the maternal lineage of individuals based on diagnostic mitochondrial SNPs with the proportion of nuclear SNPs associated with hybridizing species to evaluate the directionality of hybridization.

## Methods

### Literature review

We used Google Scholar to search for papers on hybridization involving cutthroat trout using the keywords “cutthroat trout” or “*Oncorhynchus clarkii*” and “introgression” or “hybridization,” followed by a search of the reference list of those works. Those papers providing levels of admixture attributable to individual fish (either in tables or in figures depicting hybrid indices; Jiggins and Mallet 2000) from each sample location were considered. If multiple studies were based on the same data, only data in the original study were used or we noted when some sites were repeated. We regarded a sample to potentially constitute a hybrid swarm if all fish showed some level of admixture. We accepted the definition of a nonintrogressed or parental individual used by the authors of each study. In most cases, this meant that only alleles diagnostic for one species were present in a fish. A few authors permitted slight deviations from this standard to allow for local homoplasies (Wiens and Servedio 2000) or ancient hybridization (Brown et al. 2004) or defined genotypes probabilistically (e.g., >95% probability of being a nonadmixed individual based on results from assignment tests). We acknowledge that, particularly in studies relying on relatively few diagnostic markers, the number of hybridized fish will be underestimated because some slightly hybridized fish will be overlooked (Boecklen and Howard 1997; Bennett et al. 2010). Although some authors conducted tests of Hardy–Weinberg proportions or gametic disequilibrium that are relevant to determining allele frequency distributions, we opted not to include them. Results of these analyses were not available for all locations, and when present, whether the adjustment for multiple pairwise tests was correctly applied was uncertain (Sunnucks and Hansen 2013). Moreover, for low levels of introgression or those lacking both parental forms, these are weak statistical tests unless samples sizes of individuals are very large (Boyer et al. 2008).

### Field sampling

**Figure 1:**
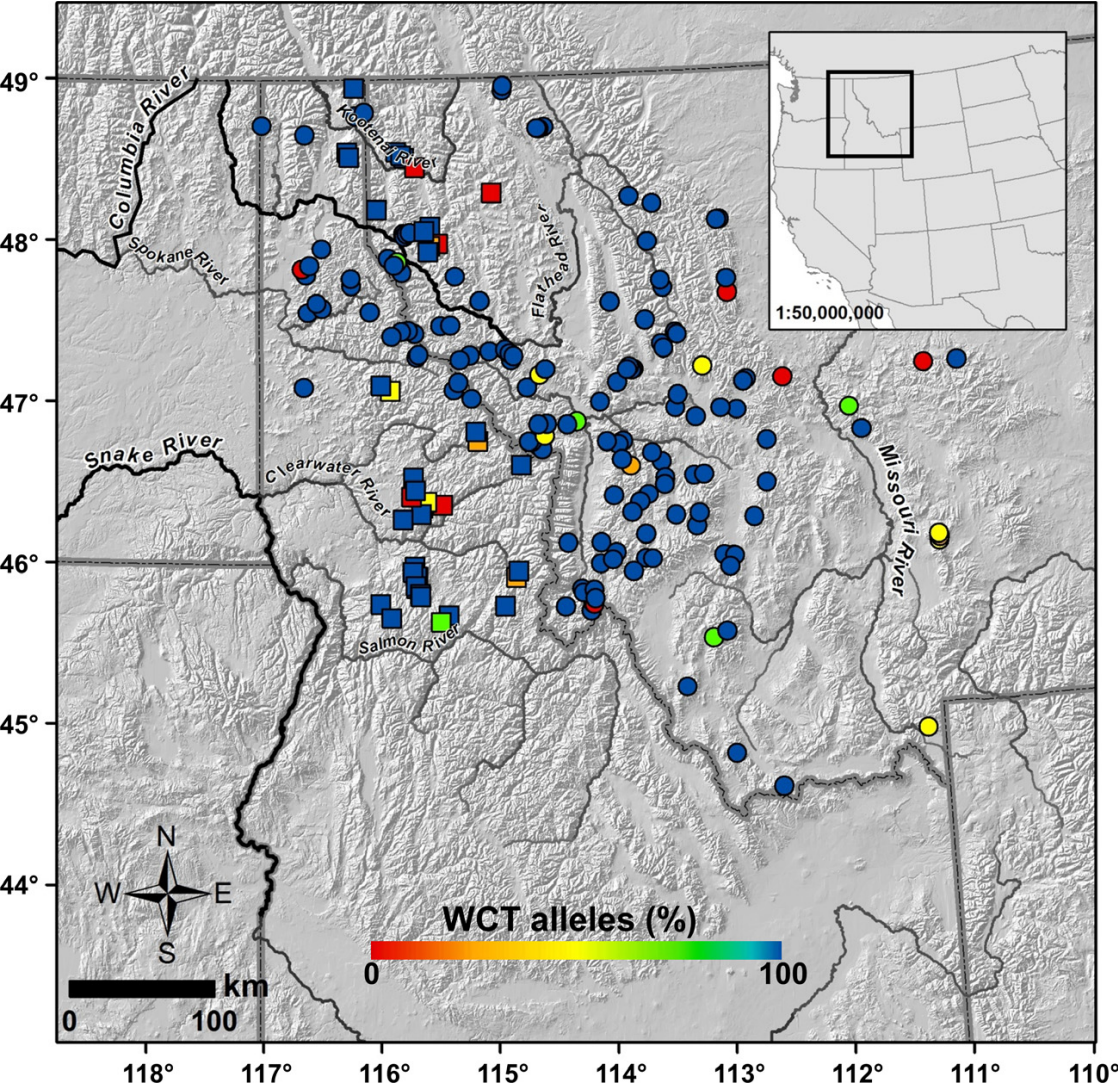
Locations where fish were genotyped in northern Idaho and western Montana. Colors are associated with the proportion of westslope cutthroat trout alleles at a site. Symbols indicate areas inside (square) or outside (circles) the historical range of rainbow trout.

Fish to be genotyped were chosen from individuals captured by electrofishing at 859 sites in 399 streams sampled from 2008 to 2012 on state and federal lands in the upper Columbia and Missouri River basins in northern Idaho and western Montana within the historical range of westslope cutthroat trout (Fig. 1). Sampled streams formed part of the PACFISH/INFISH Biological Opinion Effectiveness Monitoring Program network (PIBO; Kershner et al. 2004). This network comprises a random sample (Stevens and Olsen 1999) of about one‐third of all 6th‐code sub‐basins (area, 40–160 km^2^; Wang et al. 2011) with substantial federal ownership. Sites represented different positions within each stream. Most PIBO sites consisted of the lowermost, low‐gradient stream reach on public land (Kershner et al. 2004). In many streams, we also sampled a headwater site immediately below the confluence of a stream's uppermost perennial first‐order channels, a location approximating the upstream limit of fish presence. In a few streams, we sampled a mid‐elevation site between the PIBO and headwater sites or a site farther downstream from the PIBO site, and in one stream at two headwater locations divided by a waterfall. Captured fish were held briefly in buckets containing stream water. Before releasing them, we retained upper caudal fin clips (on chromatography paper; LaHood et al. 2008) of up to 30 *Oncorhynchus* spp. specimens captured at each site. Because we limited electrofishing at each site to 90 minutes, some samples had fewer fish. All collections were made under scientific collection permits issued (to MKY) by Montana Fish, Wildlife and Parks, and the Idaho Department of Fish and Game. All tissue specimens and extracted DNA were vouchered at the National Genomics Center for Wildlife and Fish Conservation, Missoula, MT.

### Laboratory analyses

We extracted DNA from fin clips using the Qiagen 96 DNeasy Blood and Tissue Kit following the manufacturer's instructions (QIAGEN, Valencia, CA). DNA was amplified on 96.96 Dynamic Arrays (hereafter 96.96 arrays) using the Fluidigm IFC Controller and FC1 Cycler (Fluidigm, San Francisco, CA). We used Competitive Allele Specific PCR (KASPar) assays (KBiosciences, Hoddesdon, Herts, UK) to amplify SNP loci. The PCR touchdown profile contained an initial annealing temperature of 65°C and decreased by 0.80°C per cycle until the bulk of the cycles ran at 57°C. We visualized PCR products on an EP1 Reader (Fluidigm) and determined individual genotypes using Fluidigm SNP Genotyping Software.

Candidate loci were chosen from those previously identified as being diagnostic for westslope cutthroat trout, Yellowstone cutthroat trout, or rainbow trout (Harwood and Phillips 2011; Hohenlohe et al. 2011; Kalinowski et al. 2011; Amish et al. 2012; Campbell et al. 2012; Pritchard et al. 2012). We rescreened these SNPs, eliminating those that were disproportionately indicative of introgression, that is, >2.0 SD from the mean frequency of admixture across all loci (0.078 ± 0.068). Although this pattern has been considered as evidence of extreme positive selection for nonindigenous alleles (Fitzpatrick et al. 2010), a more parsimonious explanation is that the original interpretation of such alleles as diagnostic resulted from ascertainment bias in the sample of fish used to identify those markers (Rosenblum and Novembre 2007). We further screened SNPs based on their amplification success, discarding those with failure rates >15%. To ensure accurate and consistent genotyping, each 96.96 array included five controls: two no‐DNA templates (replaced with AE buffer and H_2_O) and one of each possible F_1_ hybrid (i.e., *O. c. lewisi* × *O. mykiss*,* O. c. lewisi* × *O. c. bouvieri*, and *O. mykiss* × *O. c. bouvieri*; fish provided by Montana Fish, Wildlife & Parks’ Washoe Park Trout Hatchery). We excluded any fish if genotyping success across all SNPs was <85%. We tested for variability in SNP call rates by genotyping 81 wild fish twice. We also analyzed variability in the repeated genotyping runs of known F_1_ crosses (*n *=* *56 runs overall).

### Statistical analysis

We calculated the proportion of alleles diagnostic for westslope cutthroat trout, rainbow trout, and Yellowstone cutthroat trout in each individual. For fish with alleles from all three taxa, we used CLARKI (Kalinowski 2010) to estimate levels of admixture from each taxon. In light of genotyping error rates and the potential for local homoplasies, we regarded fish as nonintrogressed parental forms if ≥99% of alleles were from a single species (cf. Henderson et al. 2000; Hitt et al. 2003; Ostberg and Rodriguez 2004). Similarly, fish with 49–51% of alleles from each parent and with nearly all loci heterozygous were regarded as first‐generation (F1) hybrids.

We assessed whether sites represented hybrid swarms by determining whether parental forms were present and by examining the distribution of alleles. For all sites that contained hybrid fish and which had sample sizes of at least 10, we tested whether species‐diagnostic alleles were randomly distributed among individuals by comparing the allele frequencies of individual fish with the expected probability distribution of alleles based on the sample‐level proportion of admixture and assuming alleles were randomly distributed (Boyer et al. 2008). To assess departures from Hardy–Weinberg proportions in populations with admixed fish, we used GENEPOP 4.3 (Rousset 2008) to calculate *F*_IS_ and determine the number of loci exhibiting a significant heterozygote deficit or excess. Within a site, we considered genotypes to be out of Hardy–Weinberg proportions if the number of significant comparisons exceeded 5% of the number of loci examined (Waples 2015). We used logistic regression to examine the association between nuclear and mitochondrial introgression in westslope cutthroat trout × rainbow trout hybrids (excluding parental individuals and those introgressed with Yellowstone cutthroat trout). Fish from the lower McCormick Creek site were excluded from this analysis because the majority, despite being of different age classes, appeared to be F_1_ hybrids and may have been the result of ongoing stocking of F_1_ hybrids (see below). We compared levels of admixture at the upstream‐most and downstream‐most sites within streams using paired *t*‐tests, and levels of admixture inside and outside the historical range of rainbow trout using *t*‐tests assuming equal variances.

## Results

### Literature review

We found 29 studies that provided data suitable for determining whether parental forms of cutthroat trout or rainbow trout were present in a sample (Table 1). Hybridization was widespread, regardless of whether samples were from locations where one or more taxa were introduced or all were native. Moreover, sites lacking parental forms were rare. Of the 254 samples representing admixed populations, only 13 did not have parental forms and 10 of these 13 samples represented hybridization between a native cutthroat trout and nonnative Yellowstone cutthroat trout or three‐way hybrids that also included rainbow trout.

### Field locations and genotyping error rates

We removed four SNPs that appeared to be nondiagnostic, two that amplified poorly, and one that was perfectly linked with another SNP. This resulted in a set of 86 nuclear SNPs diagnostic for westslope cutthroat trout (*n *=* *35), Yellowstone cutthroat trout (*n *=* *18), and rainbow trout (*n *=* *33), and two mitochondrial SNPs diagnostic for the mitotype of each taxon (Supplemental Table S1).

Collectively, we genotyped specimens of *Oncorhynchus* from 188 locations – 91 PIBO sites, 21 mid‐elevation sites, 73 headwater sites, and three low‐elevation sites below a PIBO site – in 129 streams (Fig. 1). In 42 streams, we sampled fish at two sites, and in eight streams, three sites. We obtained genotypes from 3865 fish (*n *=* *2–30 fish/site; Table 2). Consistency in SNP call rates was high. Of wild fish genotyped twice (*n *=* *81), mean differences in call rates between genotyping runs averaged 0.19% allele changes per 172 alleles genotyped (range 0–2.09%). Variation in call rates among known crosses was also small (*n *=* *56, mean = 0.45%, range 0–3.47%).

**Table 2:** Distribution of parental fish and alleles of westslope cutthroat trout (WCT), rainbow trout (RT), and Yellowstone cutthroat trout (YCT) at sites in western Montana and northern Idaho outside and inside the historical range of rainbow trout. Sites are sorted based on increasing percentage of WCT alleles. Site refers to the PIBO site, mid‐elevation site (Middle), headwater site, or site downstream of a PIBO site (Low). Under binomial, Y indicates that proportions of parental alleles in the sample were consistent with binomial expectations, N that they were not, and NA that a site was not analyzed because the sample contained no admixed fish or was represented by fewer than 10 fish. Under loci, each entry denotes the number of loci used to test for significant heterozygote deficits (+, positive *f*_is_) or heterozygote excesses (−, negative *f*_is_) and varies depending on the parental alleles present in admixed individuals (WCT × RT, 68 loci; WCT × YCT, 53 loci; WCT × RT × YCT, 86 loci)

Stream	Site	Binomial	*N*	Parentals		Alleles (%)			*F*_IS_		
				WCT	RT	WCT^a^	RT	YCT	Loci	+	−
Rainbow trout introduced											
SF Dearborn	Low	NA	7	0	1	2.7	97.3	0	–	–	–
Blacktail Gulch	PIBO	N	30	0	17	3	96.7	0.3	86	3	0
Logging	PIBO	NA	8	0	4	7.1	92.9	0	–	–	–
Hayden	PIBO	N	17	1	12	12.9	87.1	0	68	3	0
Overwhich	Headwater (2)	Y	12	0	0	18.7	4.4	76.8	86	2	0
Welcome	PIBO	N	25	1	4	28.4	71.6	0	68	2	0
SF Sixteenmile	Middle	N	28	4	0	38.2	60.4	1.5	86	62	1
Red Canyon	Headwater	N	30	0	0	43.6	30.5	25.9	86	5	22
Lake	Headwater	N	10	2	2	44.5	55.5	0	68	3	0
SF Sixteenmile	Low	N	26	5	0	46.15	51.05	2.85	86	56	1
McCormick	PIBO	N	29	0	1	49.8	50.2	0	68	2	59
SF Sixteenmile	PIBO	N	14	1	0	54.4	39.9	5.6	86	12	0
Cloudburst	PIBO	N	30	0	0	58.8	41.2	0	68	7	16
O'Brien	PIBO	N	30	1	0	63.3	36.7	0	68	2	3
Beaver	Middle	N	10	0	1	66	34	0	68	3	0
SF Marten	PIBO	N	13	4	0	70.6	29.4	0	68	10	0
Wise	Headwater	Y	10	0	0	79.7	0.3	20	86	1	0
EF Lolo	PIBO	N	20	5	0	84.9	15.1	0	68	9	0
EF Lolo	Middle	N	10	4	0	84.9	15.1	0	68	0	0
Blacktail Gulch	Headwater	NA	3	0	0	87.7	0	12.3	–	–	–
Spring Gulch	Headwater	N	20	9	0	87.8	12.2	0	68	0	0
Chamberlain	Low	N	30	15	0	88.8	11.1	0.1	86	3	0
Grouse	PIBO	N	30	3	0	89.2	10.8	0	68	0	0
Rock	Headwater	N	30	3	0	90.1	8.7	1.2	86	4	0
EF Steamboat	PIBO	N	30	11	2	90.2	9.8	0	68	59	0
Overwhich	Headwater (1)	N	20	17	0	90.5	0.3	9.3	86	33	0
Moon	PIBO	N	18	12	0	91.7	8.3	0	68	4	0
EF Lolo	Headwater	N	10	5	0	92	8	0	68	1	0
Magpie	Middle	N	10	1	0	92	8	0	68	0	0
SF Lost Horse	PIBO	N	30	3	0	92.1	7.7	0.2	86	0	0
Hayden	Headwater	N	30	19	0	92.4	7.6	0	68	0	0
Sourdough	PIBO	N	27	22	0	92.4	3.8	3.8	86	31	0
Stony	PIBO	N	28	18	0	92.8	7.1	0.1	86	3	0
Warm Springs	PIBO	N	30	25	0	94.3	5.7	0	68	1	0
SF Petty	PIBO	N	30	21	0	95	5	0	68	0	0
Sawmill	PIBO	Y	10	0	0	95.8	0.7	3.5	86	2	0
Gold	Headwater	N	27	18	0	97.1	0.1	2.8	86	2	0
NF Dry Cottonwood	PIBO	Y	29	8	0	97.3	0.8	1.9	86	1	0
Schwartz	PIBO	N	30	27	0	97.3	2.7	0	68	3	0
Bent	PIBO	N	10	7	0	97.4	0.3	2.3	86	0	0
Foster	PIBO	Y	30	24	0	97.6	2.4	0	68	1	0
North	PIBO	Y	13	4	0	97.7	1.4	0.9	86	0	0
Second	PIBO	Y	22	15	0	97.9	2.1	<0.1	86	1	0
Sawmill	PIBO	Y	18	5	0	98.2	1.8	0	68	0	0
North	Headwater	Y	28	11	0	98.2	1.8	0	68	0	0
Marten	Middle	NA	4	3	0	98.2	1.8	0	–	–	–
Norton	PIBO	Y	30	18	0	98.3	1.7	0	68	0	0
Grouse	Headwater	NA	4	2	0	98.3	1.7	0	–	–	–
St. Regis	PIBO	N	11	9	0	98.4	1.6	0	68	0	0
SF Coal	PIBO	NA	8	5	0	98.6	1.4	0	–	–	–
Scotchman Gulch	PIBO	N	26	23	0	98.6	1.4	0	68	1	0
Sleeping Child	PIBO	N	30	29	0	98.6	1.4	0	68	1	0
SF Marten	Headwater	N	29	26	0	98.6	1.3	0.1	86	2	0
Twentyfivemile	Middle	Y	30	18	0	98.9	1.1	0	68	0	0
O'Brien	Headwater	N	30	29	0	99	1	0	68	0	0
Jerry	Headwater	Y	10	8	0	99	0.8	0.2	86	0	0
Sheep	Middle	Y	10	8	0	99.2	0.8	0	68	0	0
Rye	PIBO	N	28	25	0	99.2	0.8	0	68	0	0
Pack	PIBO	N	10	8	0	99.25	0.25	0.5	86	0	0
Rock	PIBO	N	30	25	0	99.3	0.6	0.1	86	1	0
WF Trout	PIBO	Y	30	23	0	99.3	0.7	0	68	0	0
Rock	Middle	Y	30	24	0	99.4	0.6	0	68	2	0
Beefstraight	PIBO	Y	30	22	0	99.4	0.6	0	68	0	0
NF Dupuyer	Headwater	Y	20	16	0	99.4	0.6	0	68	0	0
Sawmill	Headwater	Y	30	23	0	99.5	0.2	0.3	86	0	0
Grave	Headwater	NA	2	2	0	99.6	0.4	0	–	–	–
Siegel	PIBO	NA	2	2	0	99.6	0.4	0	–	–	–
Foster	Headwater	N	30	29	0	99.7	0	0.3	53	0	0
Stony	Headwater	Y	27	25	0	99.7	0.1	0.3	86	0	0
St. Regis	Middle	Y	14	13	0	99.7	0.3	0	68	0	0
St. Joe	Headwater	Y	29	26	0	99.7	0.1	0.2	86	1	0
WF Fishtrap	Headwater	Y	30	26	0	99.7	0.2	<0.1	86	0	0
Thayer	Headwater	NA	10	10	0	99.7	0.2	0.1	–	–	–
Snowbank	Headwater	Y	13	12	0	99.8	0	0.2	53	0	0
Moose Meadows	PIBO	NA	27	27	0	99.8	0.1	<0.1	–	–	–
Took	PIBO	NA	10	10	0	99.8	0.2	0	–	–	–
Ninemile	Headwater	Y	23	22	0	99.8	0.2	0	68	0	0
Little Joe	PIBO	NA	3	3	0	99.8	0.2	0	–	–	–
Simmons	Headwater	Y	29	27	0	99.8	0.2	0	68	0	0
Plant	PIBO	Y	10	9	0	99.8	0.2	0	68	0	0
Tyler	Headwater	NA	10	10	0	99.8	0.2	0	–	–	–
NF Dupuyer	PIBO	Y	23	22	0	99.8	0.2	0	68	0	0
4th of July	PIBO	Y	30	29	0	99.9	0.1	0	68	0	0
Grave	Middle	Y	12	11	0	99.9	0.1	0	68	0	0
Jim	PIBO	NA	15	15	0	99.9	<0.1	0	–	–	–
Snowbank	PIBO	Y	29	28	0	99.9	<0.1	0.1	86	0	0
NF Lower Willow	Headwater	NA	30	30	0	99.9	0.1	0	–	–	–
SF Willow	PIBO	NA	18	18	0	99.9	<0.1	0	–	–	–
SF Willow	Headwater	NA	30	30	0	99.9	0.1	0	–	–	–
Snowshoe	PIBO	NA	30	30	0	99.9	<0.1	0	–	–	–
Beefstraight	Headwater	Y	30	29	0	99.9	0.1	0	68	0	0
Overwhich	Middle	NA	22	22	0	99.9	0.1	0	–	–	–
EF Bitterroot	PIBO	Y	28	27	0	99.9	0.1	0	68	0	0
Warm Springs	Headwater	NA	23	23	0	99.9	<0.1	0	–	–	–
Sleeping Child	Headwater	Y	30	29	0	99.9	0.1	0	68	2	0
Burnt Fork Bitterroot	PIBO	NA	30	30	0	99.9	0.1	0	–	–	–
McCormick	Headwater	Y	27	26	0	99.9	0.1	0	68	0	0
Little Joe	Middle	NA	18	18	0	99.9	0.1	0	–	–	–
Zero	PIBO	NA	10	10	0	99.9	0.1	0	–	–	–
Siegel	Middle	NA	30	30	0	99.9	<0.1	0	–	–	–
McElwain	PIBO	NA	10	10	0	99.9	0.1	0	–	–	–
Wasson	Headwater	NA	10	10	0	99.9	0.1	0	–	–	–
Rye	Headwater	NA	13	13	0	99.9	0.1	0	–	–	–
Skin	PIBO	NA	28	28	0	99.9	<0.1	0	–	–	–
Ramskull	PIBO	Y	20	19	0	99.9	0.1	0	68	1	0
West Gold	PIBO	NA	25	25	0	100	0	0	–	–	–
Armstrong	PIBO	NA	10	10	0	100	0	0	–	–	–
4th of July	Headwater	NA	29	29	0	100	0	0	–	–	–
WF Rock	Middle	NA	10	10	0	100	0	0	–	–	–
SF Coal	Middle	NA	10	10	0	100	0	0	–	–	–
SF Coal	Headwater	NA	4	4	0	100	0	0	–	–	–
Lewis	PIBO	NA	2	2	0	100	0	0	–	–	–
Willow	Middle	NA	10	10	0	100	0	0	–	–	–
NF Lower Willow	PIBO	NA	23	23	0	100	0	0	–	–	–
SF Douglas	PIBO	NA	30	30	0	100	0	0	–	–	–
Little Blackfoot	PIBO	NA	12	12	0	100	0	0	–	–	–
Copper	PIBO	NA	10	10	0	100	0	0	–	–	–
MF Rock	PIBO	NA	19	19	0	100	0	0	–	–	–
Schwartz	Headwater	NA	3	3	0	100	0	0	–	–	–
Piquett	PIBO	NA	4	4	0	100	0	0	–	–	–
Piquett	Middle	NA	10	10	0	100	0	0	–	–	–
Sheridan	Middle	NA	10	10	0	100	0	0	–	–	–
SF Petty	Headwater	NA	29	29	0	100	0	0	–	–	–
Slowey	Headwater	NA	10	10	0	100	0	0	–	–	–
Savenac	Headwater	NA	9	9	0	100	0	0	–	–	–
Twelvemile	Headwater	NA	10	10	0	100	0	0	–	–	–
St. Regis	Headwater	NA	25	25	0	100	0	0	–	–	–
Bird	PIBO	NA	30	30	0	100	0	0	–	–	–
Bird	Headwater	NA	30	30	0	100	0	0	–	–	–
Red Ives	Headwater	NA	10	10	0	100	0	0	–	–	–
Mokins	Headwater	NA	18	18	0	100	0	0	–	–	–
Siegel	Headwater	NA	30	30	0	100	0	0	–	–	–
Weeksville	Headwater	NA	10	10	0	100	0	0	–	–	–
Chamberlain	PIBO	NA	30	30	0	100	0	0	–	–	–
NF St. Joe	Headwater	NA	14	14	0	100	0	0	–	–	–
Helen	PIBO	NA	10	10	0	100	0	0	–	–	–
Second	Headwater	NA	30	30	0	100	0	0	–	–	–
NF Gold	PIBO	NA	30	30	0	100	0	0	–	–	–
EF Steamboat	Headwater	NA	30	30	0	100	0	0	–	–	–
Skin	Headwater	NA	30	30	0	100	0	0	–	–	–
Welcome	Headwater	NA	4	4	0	100	0	0	–	–	–
Marshall	Headwater	NA	10	10	0	100	0	0	–	–	–
Youngs	Headwater	NA	10	10	0	100	0	0	–	–	–
Willow	Headwater	NA	10	10	0	100	0	0	–	–	–
Otter	PIBO	NA	17	17	0	100	0	0	–	–	–
Otter	Headwater	NA	10	10	0	100	0	0	–	–	–
Rainbow trout native											
Hungery	PIBO	Y	20	0	15	0.6	99.4	0	68	0	0
Little Wolf	PIBO	Y	28	0	12	1.6	98.4	<0.1	86	0	0
Lolo	PIBO	N	15	0	10	1.8	98.2	0	68	0	0
Bobtail	PIBO	Y	10	0	6	2.3	97.7	0	68	2	0
Silver Butte Fisher	PIBO	N	15	1	3	8.7	91.3	0	68	57	0
Osier	PIBO	N	14	3	10	21.6	78.4	0	68	68	0
Bad Luck	PIBO	N	30	7	20	26.6	73.4	0	68	68	0
Silver Butte Fisher	Middle	N	12	2	0	30.9	69.1	0	68	34	0
Hungery	Headwater	N	30	5	6	43.8	56.2	0	68	67	0
Little NF Clearwater	PIBO	N	16	4	3	54.9	45.1	0	68	43	0
Newsome	PIBO	N	28	15	2	62.2	37.8	0	68	67	0
SF Red	Headwater	N	28	17	0	72.7	27.3	0	68	67	0
Flat	PIBO	N	28	19	0	84.5	15.5	0	68	12	0
SF Red	PIBO	N	18	15	1	85.1	14.9	0	68	65	0
Baldy	PIBO	N	30	22	3	87.1	12.9	0	68	68	0
O'Brien	Headwater	Y	22	0	0	88.7	0	11.3	53	1	3
Leggett	PIBO	N	27	21	1	89.6	10.4	0	68	66	0
Leggett	Headwater	N	28	20	0	95.6	4.4	0	68	2	0
Miller	PIBO	Y	26	4	0	95.7	4.3	0	68	1	0
Fish	Headwater	N	10	9	0	96.4	3.6	0	68	0	0
Bad Luck	Headwater	N	30	29	1	96.4	3.6	0	68	68	0
Silver Butte Fisher	Headwater	N	30	27	0	96.9	3.1	0	68	3	0
Moores Lake	PIBO	N	30	19	0	97.6	2.4	0	68	1	0
Boulder	PIBO	Y	30	12	0	98	1.8	0.3	86	0	0
American	PIBO	Y	10	1	0	98.6	1.4	0	68	0	0
Little NF Clearwater	Headwater	Y	20	11	0	99	1	0	68	0	0
French	PIBO	Y	29	20	0	99	1	0	68	0	0
WF Quartz	PIBO	Y	21	15	0	99.2	0.8	0	68	1	0
Osier	Headwater	Y	29	24	0	99.4	0.6	0	68	0	0
Santiam	PIBO	Y	30	23	0	99.4	0.6	0.1	86	0	0
Santiam	Headwater	Y	29	25	0	99.5	0.5	0	68	1	0
Lolo	Headwater	Y	29	26	0	99.6	0.4	0	68	0	0
West Fisher	Middle	Y	30	27	0	99.7	0.3	<0.1	86	0	0
Newsome	Headwater	Y	30	29	0	99.7	0.3	0	68	0	0
Baldy	Headwater	Y	27	26	0	99.8	0.2	0	68	0	0
WF Quartz	Headwater	Y	29	28	0	99.8	0.2	0	68	0	0
Mud	PIBO	NA	23	23	0	99.8	0.2	0	–	–	–
Shotgun	PIBO	NA	30	30	0	99.8	0.2	0	–	–	–
Boulder	Headwater	NA	30	30	0	99.9	0.1	0	–	–	–
Canyon	PIBO	NA	10	10	0	99.9	0.1	0	–	–	–
Spruce	PIBO	NA	10	10	0	100	0	0	–	–	–
Ross	Middle	NA	30	30	0	100	0	0	–	–	–

a If alleles from all three taxa were present, the proportional contribution of each to the overall level of hybridization was estimated using program CLARKI (Kalinowski 2010); the number of significant digits in these columns is limited by the precision of this estimate.

### Introgression among individuals and sites

**Figure 2:**
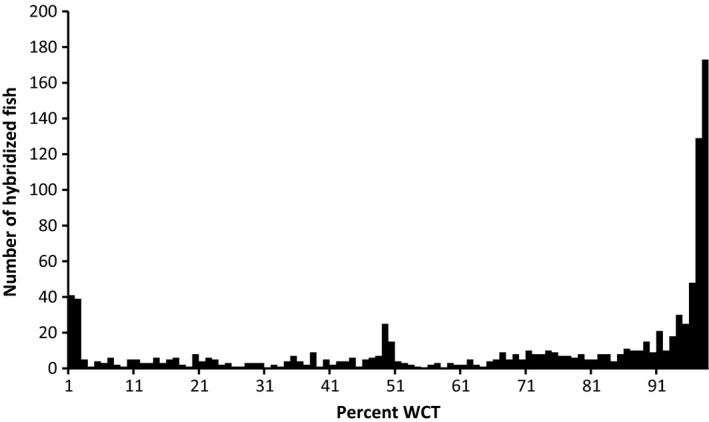
The frequency of levels of admixture of nonparental fish (*n *=* *957) based on the percentage of westslope cutthroat trout (WCT) alleles in each fish.

The majority of genotyped fish represented parental forms (Table 2). Nearly 72% of sampled fish were parental westslope cutthroat trout, and 3.5% were parental rainbow trout; there were no parental Yellowstone cutthroat trout. Of the 137 parental rainbow trout sampled, 68% were from areas where rainbow trout are native. There were 957 admixed fish representing 25% of the sample; over 81% were westslope cutthroat trout × rainbow trout crosses, less than 9% were westslope cutthroat trout × Yellowstone cutthroat trout crosses, and the rest (10%) had alleles from all three taxa. A minority (41%) of hybrid fish had >10% admixture (Fig. 2). First‐generation hybrids between westslope cutthroat trout and rainbow trout were rare (*n *=* *33), and 15 of these came from a single location (McCormick Creek PIBO).

Although admixed fish were present at most of the sites, levels of admixture tended to be low. Of the 188 sites sampled, 73% contained ≥95% westslope cutthroat trout alleles, 58% had ≥99% westslope cutthroat trout alleles, and 37% contained only parental westslope cutthroat trout (Table 2, Fig. 1). An additional six sites were nearly pure (≥95%) rainbow trout; four of these were in areas where rainbow trout are native.

### Hybrid swarms

Samples meeting the criteria for hybrid swarms – no parental fish, a random distribution of alleles among sampled fish, and sampled genotypes in Hardy–Weinberg proportions – were rare and were associated with the presence of Yellowstone cutthroat trout alleles (Table 2). Parental westslope cutthroat trout were present at nearly 90% of all sites. Parental rainbow trout were present at 12% of sites, but always accompanied by parental westslope cutthroat trout, hybrids, or both. Seven sites were represented only by hybridized fish; at six of these sites, Yellowstone cutthroat trout alleles constituted a large proportion of the sample (mean 24.9%, range 3.5–76.8%). Of the 113 sites with at least one admixed fish and at least 10 fish sampled, 60 had allelic distributions with significant deviations from binomial expectations. At the 44 sites that had ≥5% nonwestslope cutthroat trout alleles, allelic distributions in all but two deviated from binomial expectations, and both of these contained >10% Yellowstone cutthroat trout alleles (Table 2). Samples from 26 sites were not in Hardy–Weinberg proportions; at only one of these sites did allelic distributions fit binomial expectations.

**Figure 3:**
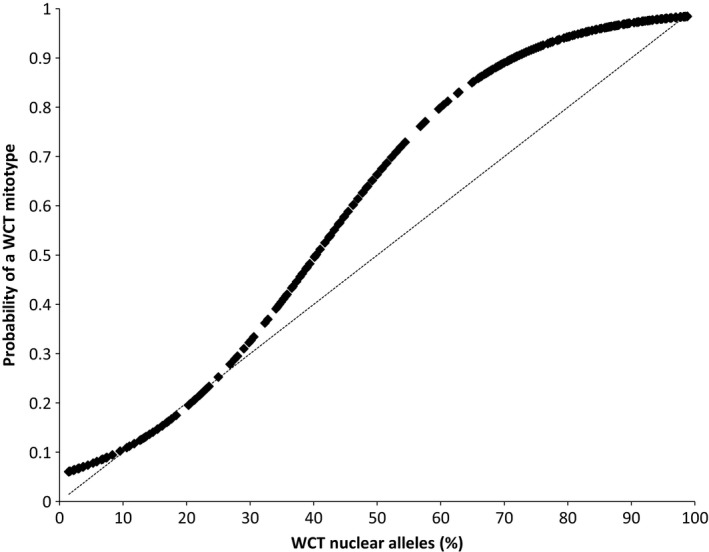
Relationship between the percentage of nuclear alleles from westslope cutthroat trout (WCT) in an individual and the probability in an individual would have a westslope cutthroat trout mitotype. The curve is described by a logistic equation with the intercept ‐2.8496 and coefficient 0.0705. A 1:1 relation (dotted line) is included for comparison.

Hybridization was bidirectional, but most fish had the mitochondrial haplotype of westslope cutthroat trout. Of the 3861 fish for which we examined mitochondrial DNA, only 0.7% had the haplotype of Yellowstone cutthroat trout and 9.4% that of rainbow trout. Among F_1_ hybrids not from the McCormick Creek PIBO site, 12 of 18 fish had a westslope cutthroat trout haplotype; all those from the McCormick Creek site had a rainbow trout haplotype. Although the proportion of nuclear alleles and the probability of having a mitochondrial haplotype were correlated in hybrids between westslope cutthroat trout and rainbow trout, these individuals were disproportionately likely to have a westslope cutthroat trout haplotype (Fig. 3). For example, individuals with 50% westslope cutthroat trout nuclear alleles had a 66% probability of having a westslope cutthroat trout haplotype. No parental westslope cutthroat trout had the haplotype of another taxon, but three fish with > 99% nuclear alleles representative of rainbow trout had a westslope cutthroat trout haplotype.

### Spatial patterns

Streams sampled at more than one site exhibited pronounced longitudinal differences in introgression. The percentage of westslope cutthroat trout alleles was significantly higher at upstream sites than at downstream sites (means, 96.3% vs. 77.7%; *n *=* *48; *P *<* *0.0001); this analysis included eight streams in which only parental fish were present at both upstream and downstream sites. In 12 of the remaining 40 streams, despite varying levels of introgression at downstream sites (mean westslope cutthroat trout alleles 88.2%, range 28.4–99.9%), only parental westslope cutthroat trout were observed at upstream sites. We excluded two streams with stocked headwater lakes (Rock and Overwich Creeks) from this analysis. In each of these, introgression substantially declined downstream from the source of stocked fish (Table 2).

Whether rainbow trout were indigenous or introduced to a location had little influence on the prevalence of parental westslope cutthroat trout, but did influence patterns of admixture. Of the 42 sites in the historical range of rainbow trout, over 88% had parental westslope cutthroat trout, but 86% had westslope cutthroat trout × rainbow trout hybrids. For headwater sites within (*n *=* *15) and outside (*n *=* *57) the historical range of rainbow trout, levels of introgression with rainbow trout were similar (means, 92.5% vs. 95.9% westslope cutthroat trout alleles among all fish at a site; *P *=* *0.35). Among low‐elevation sites, however, introgression with rainbow trout tended to be higher inside (*n *=* *24) than outside (*n *=* *68) the historical range of rainbow trout (means, 67.2% vs. 88.7% westslope cutthroat trout alleles among all fish at a site; *P *=* *0.003).

## Discussion

To our knowledge, this is among the first studies to use a large panel of diagnostic nuclear SNPs to evaluate hybridization in a broadly distributed, representative group of streams (also see Yau and Taylor 2013; Lamer et al. 2015), and the first to contrast patterns of introgression between cutthroat trout and rainbow trout inside and outside the latter's historical range. This suite of diagnostic markers allowed us to detect hybridization at extremely low levels and to evaluate the distribution of alleles within individual fish. Single nucleotide polymorphisms are subject to local homoplasy and therefore may not be universally diagnostic. We removed four SNPs based on statistical evidence that they were not diagnostic. Even after this screening, our approach most likely retained some nondiagnostic loci. Further, given the preponderance of nearly pure to pure westslope cutthroat trout, genotyping errors would, by chance, primarily have been interpreted as hybridization. Given these biasing factors, reported levels of hybridization are likely slightly inflated.

Nevertheless, we found that hybridization was absent or minor at the majority of sites and that pure parental westslope cutthroat trout were the most common genotype. We also found that few samples could be considered hybrid swarms. In those populations with both sufficient admixture (≥5%) and sample sizes (≥10) to provide meaningful tests of random mating, none of the sites lacking Yellowstone cutthroat trout alleles had allele distributions consistent with random mating and, at many of these sites, parental rainbow trout and westslope cutthroat trout were sympatric. Given the extended stocking histories associated with many of these sites, it is unlikely that these patterns represent transient phenomena; allele distributions consistent with binomial expectations can emerge in as little as five generations of random mating (Kalinowski and Powell 2015). It is also not the case that, due to processes such as within‐stream movement, drawn samples would never resemble swarms: Sites with significant numbers of alleles diagnostic for Yellowstone cutthroat trout produced samples consistent with random mating and lacking parental types. Rather, the prevalence of both parental types within a sample, the scarcity of F1 hybrids, and the preponderance of minimally hybridized fish collectively indicate that mating between parental (or nearly parental) rainbow trout and westslope cutthroat trout is rare in the streams we examined. Patterns of hybridization involving Yellowstone cutthroat trout, however, differed from those involving rainbow trout and should not be conflated.

### Introgression with rainbow trout

Although the use of a panel of 86 diagnostic nuclear markers allowed much more precise estimation of individual levels of hybridization, our findings were strikingly similar to those of previous studies, in which hybrids with rainbow trout were found at most sites (mean among studies 67%, range 50–78%), yet most fish were nonadmixed cutthroat trout (mean 77%, range 58–90%), regardless of whether rainbow trout were indigenous to a basin (Weigel et al. 2003; Kozfkay et al. 2007; Williams et al. 2007; Loxterman et al. 2014) or introduced (Rubidge and Taylor 2004; Boyer et al. 2008; Gunnell et al. 2008; Bennett and Kershner 2009, 2009; Kovach et al. 2011; Rasmussen et al. 2012; Yau and Taylor 2013). Given that we observed somewhat greater levels of introgression in areas where rainbow trout were native, we conclude that there is little geographic contribution to the strength of reproductive barriers between these species, regardless of whether secondary contact occurred during the invasion of the Columbia River basin by rainbow trout during the Pleistocene (Behnke 1992) or more recently via human agency. We acknowledge, however, that stocking of rainbow trout has been so widespread that geographic differences may be obscured. Regardless, introgression of rainbow trout alleles into populations of westslope cutthroat trout is common.

Another point of agreement among most studies of introgression between cutthroat trout and rainbow trout is that first‐generation hybrids make up a small percentage of introgressed fish, for example, 3.6% of hybrid fish in this study and 1.5–5.5% elsewhere (Rubidge and Taylor 2004; Baumsteiger et al. 2005; Ostberg and Rodriguez 2006; Gunnell et al. 2008; Rasmussen et al. 2012). This suggests that parental types of these species rarely hybridize, perhaps because of strong assortative mating (Mallet 2005), for example, cutthroat trout generally spawn in smaller streams later in the year after flows have peaked (Hartman and Gill 1968; Magee et al. 1996; DeRito et al. 2010). But spawning times and locations occasionally overlap, facilitated in part by salmonid males that remain capable of spawning for an extended period and search for females during the spawning season (Neville et al. 2006; Muhlfeld et al. 2009b). As has been previously observed (Rubidge and Taylor 2004; Kozfkay et al. 2007), parental crosses were bidirectional and generally tended to favor rainbow trout males and westslope cutthroat trout females. Westslope cutthroat trout haplotypes were disproportionately prevalent in later‐generation hybrids, for which the simplest explanation is that this taxon was more abundant in the tributaries we sampled. Nonetheless, the association between nuclear and mitochondrial genotypes, and the relative rarity of moderately hybridized fish, indicates strong assortative mating such that hybrid fish tended to associate with like individuals, for example, fish having primarily westslope cutthroat trout alleles tended to reproduce with similar, or less admixed, individuals (cf. Bettles et al. 2005).

A third point of consistency is the decline in admixture of westslope cutthroat trout populations in the upper reaches of a basin. Longitudinal zonation in streams, with cutthroat trout upstream from rainbow trout, is typical (Fausch 1989; Bozek and Hubert 1992; Meyer et al. 2006) and can arise regardless of the location of stocking of parental populations (Paul and Post 2001). These upstream–downstream differences are common to virtually every study of introgression between cutthroat trout and rainbow trout (e.g., Ostberg et al. 2004; Gunnell et al. 2008; Rasmussen et al. 2010; Muhlfeld et al. 2014) and are even evident where nonnative westslope cutthroat trout have been introduced to streams with indigenous populations of rainbow trout (Neville and Dunham 2011). Some have considered the upstream–downstream differences to represent a snapshot of the ongoing advance of alleles from (usually) downstream sources of rainbow trout, based on the apparent spread of introgression over time (Rubidge et al. 2001; Hitt et al. 2003; Muhlfeld et al. 2014). But this trend is not universal; introgression between rainbow trout and cutthroat trout has remained stable or even declined in some locations (Ostberg and Rodriguez 2006; Williams et al. 2007; Bennett and Kershner 2009; Rasmussen et al. 2010; Ostberg and Chase 2012; Buehrens et al. 2013), albeit over relatively short intervals (one to several years). That many hybrid zones may be relatively stable is implicit given the number of generations needed to produce slightly introgressed fish (Kanda et al. 2002; Gunnell et al. 2008). Moreover, it is highly implausible that streams throughout the northwestern United States would show similar longitudinal patterns if introgressive invasion by rainbow trout was rapid and ongoing (cf. Kanda et al. 2002). Collectively, this suggests that introgression between rainbow trout and cutthroat trout is influenced by propagule pressure (from either wild populations or stocking), environmental variables (Al‐Chokhachy et al. 2014; Muhlfeld et al. 2014), or both (Weigel et al. 2003; Ostberg et al. 2004; Heath et al. 2010; Bennett et al. 2010; Rasmussen et al. 2010; Loxterman et al. 2014; Yau and Taylor 2013). Among stream fishes (and many other taxa), environmental mediation of hybrid zones may be the norm (Jiggins and Mallet 2000; Keller and Seehausen 2012; Culumber et al. 2014).

The large reservoir of parental westslope cutthroat trout at many headwater locations also suggests that their status may be less dire than previously thought. Shepard et al. (2005) estimated that nearly 2/3 of all habitats occupied by westslope cutthroat trout featured hybrid fish, but considered the detection of hybrids at any site in a stream to be indicative of their presence throughout a stream. We suspect that comprehensive sampling of headwater sites, which represent the bulk of habitat in dendritic stream networks, would reveal many stream segments composed primarily, or entirely, of nonintrogressed fish. In our sample, of the 48 streams we sampled longitudinally, 16% of the samples were not introgressed at the downstream sites compared with 41% at the headwaters sites. Although the populations typical of headwaters sites are often thought to be at risk because of their size and location, westslope cutthroat trout appear to persist in extremely small habitats (Peterson et al. 2014) and many of these waters may constitute climate refugia for cutthroat trout in coming decades (Isaak et al. 2015). Their presence at these sites, combined with the mobility often exhibited by cutthroat trout in small streams (Young 2011), also implies that removal of sources of nonnative rainbow trout alleles, by reduction of naturalized rainbow trout populations, stocking infertile rainbow trout, or by installation of migration barriers, may lead to the reduction or even disappearance of introgressed individuals from downstream populations (Amador et al. 2012), although this has yet to be demonstrated in salmonids (High 2010).

### Introgression with Yellowstone cutthroat trout

Consistent with previous studies, westslope cutthroat trout and Yellowstone cutthroat trout form hybrid swarms. In previous studies, most (6/8) of the sites where hybridization occurred between these two species contained no parental fish (Table 1). In this study, even though we observed far fewer individuals introgressed with Yellowstone cutthroat trout than those introgressed with rainbow trout, we observed no parental Yellowstone cutthroat trout in our samples, and most samples consistent with swarms contained Yellowstone cutthroat trout alleles. Yellowstone cutthroat trout are relatively closely related to westslope cutthroat trout (Behnke 1992; Loxterman and Keeley 2012) and are similar with respect to the timing and location of spawning (Magee et al. 1996; DeRito et al. 2010). Although reproductive barriers between these taxa may be weak, longitudinal patterns in introgression were occasionally observed that might be attributable to evolutionary differences in life histories. For example, although individuals primarily composed of Yellowstone cutthroat trout alleles were dominant at a headwater site in Overwhich Creek which was above a waterfall and downstream from a headwater lake stocked with Yellowstone cutthroat trout, sites farther downstream were dominated by fish primarily or entirely composed of westslope cutthroat trout alleles. Most Yellowstone cutthroat trout historically stocked throughout the western United States were derived from fish that spawned in tributaries, but spent the majority of their lives in a lake (Gresswell and Varley 1988). Although introgression in many streams was apparent in our samples, these often had lakes in their watersheds and it may be that adaptation to lacustrine environments has precluded more widespread introgression by Yellowstone cutthroat trout. Regardless, more comprehensive inventories of the extent of introgression of introduced Yellowstone cutthroat trout with other subspecies of cutthroat trout and with native rainbow trout are warranted, especially given that naturalized populations of Yellowstone cutthroat trout outside their historical range have been mistaken for other subspecies of cutthroat trout (Pritchard et al. 2007, 2015).

## Conclusion

There is no doubt that widespread introductions of rainbow trout and Yellowstone cutthroat trout have increased the incidence of introgressive hybridization in populations of westslope cutthroat trout. Recent or ancient contact between rainbow trout and westslope cutthroat trout, however, appears to have had little effect on the structure of their hybrid zones, whereas position within a watershed had a large influence. Our ability to discern these patterns derives in part from examining many diagnostic markers within individual fish, and from broad regional sampling within streams and among river basins. But it also derives from a reassessment of the data from many studies involving these species, with an emphasis on whether those data are consistent with the interpretations that have been offered. Overall, these observations challenge the notion that hybridization between westslope cutthroat trout and rainbow trout inexorably leads to the formation of a hybrid swarm. Given that introgression has had over 100 years to spread in locations where rainbow trout have been introduced (including headwater lakes; Bennett et al. 2010) and thousands of years where they naturally co‐occur with cutthroat trout, and that even nonanadromous rainbow trout can move tens to hundreds of kilometers in a single year (Bjornn and Mallet 1964), there has been sufficient time for individuals of this species or their hybrid offspring to have reached all portions of nearly every accessible watershed in this region. Yet we found, as have many others, that parental fish were common and allele distributions often nonrandom in hybrid zones, and consequently that hybrid swarms were rare. One could regard this as a semantic argument (e.g., Kalinowski and Powell 2015) that rests on the definition of a hybrid swarm, for example, the mere presence of hybridized individuals beyond the first generation (Rhymer and Simberloff 1996) or the more restrictive conditions that we and others (Allendorf et al. 2013) have adopted. Regardless, the presumption that hybrid swarms will inevitably form following nonnative species introductions continues to influence the conservation of cutthroat trout (Allendorf et al. 2005; Campton and Kaeding 2005; Shepard et al. 2005) and of many other species (Blum et al. 2010; Bean et al. 2013; Hasselman et al. 2014). Abandoning this term might be warranted, except in a few instances (e.g., hybridization involving *Barbus barbus* in northern Italy; Meraner et al. 2013). Rather than emphasizing the uncertain threat posed by the presence of hybrid individuals, the focus should be on understanding those environmental and anthropogenic factors related to the position, structure, and dynamics of hybrid zones (Culumber et al. 2012).

## Data accessibility and supporting materials

All SNP markers used were previously published and have been archived in GenBank. Names, sequences, and primary publications for all SNP markers are found in Table S1. SNP data for the individuals used in analysis including sample locations are available as an Excel file archived in Dryad (http://datadryad.org/).

## Conflict of Interest

None declared.

## Supporting information


**Table S1**. Diagnostic single nucleotide (SNP) markers used to evaluate salmonid hybridization.
