# Supplementary material for: Patterns of hybridization among cutthroat trout and rainbow trout in northern Rocky Mountain streams
Source: Ecol Evol. 2016 Jan 11;6(3):688–706. doi: 10.1002/ece3.1887 (PMC4739558; doi:10.1002/ece3.1887)
Supplement: Supplementary file 1 — Table S1. Diagnostic single nucleotide (SNP) markers used to evaluate salmonid hybridization. [file ECE3-6-688-s001.docx]

Table S1. Diagnostic single nucleotide (SNP) markers used to evaluate salmonid hybridization. Full citations to the SNP sources can be found in the main document.

| **SNP** | **Diagnostic** | **X Allele Primer** | **Y Allele Primer** | **Common Primer** | **Source** |
| --- | --- | --- | --- | --- | --- |
| Ocl_16S-348 | mtDNA | AGATTATGCTAGAATGAGTAATAAGAGAGA | AGATTATGCTAGAATGAGTAATAAGAGAGT | CTTACACATGTGCTGGGAGAGAGTT | *Campbell et al. 2012* |
| OclWD_mtCOXW3_Har | mtDNA | GGGAATTAATCAGTTTCCAAAGCCC | GGAATTAATCAGTTTCCAAAGCCT | CGTCACAGCCCATGCCTTCGTT | *Harwood and Phillips 2011* |
| FLU_OclWD_104415L _Garza | WSCT | GCCTTGCCAGACACATTCTAGTC | CCTTGCCAGACACATTCTAGTA | GTATGCCGAAAGGATCACCTGCTTT | *Campbell et al. 2012* |
| FLU_OclWD_105897L _Garza | WSCT | ACAAATAATCCAATTTGAAATGTTTCAAAATG | CACAAATAATCCAATTTGAAATGTTTCAAAATT | CTCATCTTTGGCAACAGGGACACAT | *Campbell et al. 2012* |
| FLU_OclWD_107607L _Garza | WSCT | TGGATTCCATTGTGCAAAAGTTATATGTT | GATTCCATTGTGCAAAAGTTATATGTC | AGTGATGAGAGGTTTCCGGAAAAT | *Pritchard et al. 2011; Campbell et al. 2012* |
| FLU_OmyRD_RAD_21362_Hoh | WSCT | GCTGCTCTGCTGACGGTTC | CTGCTCTGCTGACGGTTA | GGTTGAACTGGACGAGCCGGAA | *Hohenlohe et al. 2011; Amish et al. 2012* |
| FLU_OmyRD_RAD_23910_Hoh | WSCT | TCCTGCAGGGTGTCGGCG | CTCCTGCAGGGTGTCGGCA | CGCTTTAAACAGCTGGTGGACAGTA | *Hohenlohe et al. 2011; Amish et al. 2012* |
| FLU_OmyRD_RAD_29419_Hoh | WSCT | CCCGCCAGATGGCCAGG | CCCCGCCAGATGGCCAGA | CATGGAGGACCTGAGTGCTCTAAA | *Hohenlohe et al. 2011; Amish et al. 2012* |
| FLU_OmyRD_RAD_43425_Hoh | WSCT | ATCTGTTGACTCCCTCTCCTCT | ATCTGTTGACTCCCTCTCCTCC | TTTACCAGGCGGTGCGGCAGTT | *Hohenlohe et al. 2011; Amish et al. 2012* |
| FLU_OmyRD_RAD_48390_Hoh | WSCT | TCTGCCAGTCTGTCAGGTCG | CTCTGCCAGTCTGTCAGGTCA | GATGCTGTGTGGGATGCAGGAGAT | *Hohenlohe et al. 2011; Amish et al. 2012* |
| OclWD_114315L_Garza | WSCT | AAGGGCCCATTTAGGACTGGG | AAGGGCCCATTTAGGACTGGC | TGAGTGACCCTTGACCTGTGACAT | *Campbell et al. 2012* |
| FLU_OmyRD_RAD_53822_Hoh | WSCT | AACACCGATATACATAAATGTGCTGT | AACACCGATATACATAAATGTGCTGG | GACTCAGCCTGCAGGGGTCATA | *Hohenlohe et al. 2011; Amish et al. 2012* |
| Ocl_WD_114448_Garza | WSCT | GCCGAAAGGTAAAATCCACAAATCC | ATGCCGAAAGGTAAAATCCACAAATCA | TAGGCTAACAGGAGAAGCTGACG | *Pritchard et al. 2012; Campbell et al. 2012* |
| Ocl_WD_94903L_Garza | WSCT | GAGGATCAATACATACTGCATGTTTG | GAGGATCAATACATACTGCATGTTTT | TGCGGTTTGGATCCAGCTCTCC | *Campbell et al. 2012* |
| OclRD__CLK3W4_Har | WSCT | GGCAGCACTCCACAGCAGC | GGCAGCACTCCACAGCAGA | GGTGTCTCGTAACAGGTTCTGTTGTT | *Harwood and Phillips 2011* |
| FLU_OmyRD_RAD_44764_Hoh | WSCT | TGAAGAAGCCGGATGTGGAGG | TGAAGAAGCCGGATGTGGAGA | CTCACAAGCGCAGTTCGCATGTAA | *Hohenlohe et al. 2011; Amish et al. 2012* |
| OclRD_CLK3W5_Har | WSCT | AGTCTTCAGTGCGGTCGACC | ACAGTCTTCAGTGCGGTCGACT | GAGATCATCATGGTGTTGTAGAGGGAA | *Harwood and Phillips 2011* |
| OclWD_101704L _Garza | WSCT | ATTCTCATATACACACAGCATCTCTGT | TCTCATATACACACAGCATCTCTGC | CCTTCTCTTGATCTCCTCCACTAGTT | *Pritchard et al. 2012* |
| OclWD_104216L _Garza | WSCT | ATTTGTTAGATTGTCTAGTCTTCACTGT | TTTGTTAGATTGTCTAGTCTTCACTGC | GTTCTACAAACATGGCGAGACCCAT | *Pritchard et al. 2012; Campbell et al. 2012* |
| OclWD_105075L_Garza | WSCT | GGAAAGTAGTTAATGAAAGATGTTATCCTA | AAAGTAGTTAATGAAAGATGTTATCCTG | AAGATGAAGCAGACCGTGCAGCTTT | *Campbell et al. 2012* |
| OclWD_107031L _Garza | WSCT | GTGTGGGTGGCAGAGCTGGAT | TGGGTGGCAGAGCTGGAG | CTGTGCCCTCTTGGATTAGCATGTT | *Pritchard et al. 2012; Campbell et al. 2012* |
| OclWD_127556L _Garza | WSCT | TACGGAAGTGATGTATGTAAAGGTTTC | ATTACGGAAGTGATGTATGTAAAGGTTTT | GTGCTCTGCTGGTAAAACTGTACAAATAT | *Pritchard et al. 2012; Campbell et al. 2012* |
| OclWD_129170L _Garza | WSCT | ACATTGTCACTTGATATCTTGTTACA | ACACATTGTCACTTGATATCTTGTTACT | AAACGAGGGAGCCAATGTACGTCAA | *Campbell et al. 2012* |
| OclWD_aldB_419NC | WSCT | GGCTATTCTAGGGTAGAGTGGAC | GCTATTCTAGGGTAGAGTGGAT | TACAATAGCTTCCCAATGATATCCCTGTA | *Campbell et al. 2012* |
| OclWD_P53_307Kal | WSCT | TTGACCCCCAACTAATGTCTTGTC | TTGACCCCCAACTAATGTCTTGTT | GAGAGGAAGTTCCCATCACTGTCAT | *Kalinowski et al. 2011* |
| OclWD_ppie_32NC | WSCT | GTACATGGATTGTTCCTGTTGGTTCT | TACATGGATTGTTCCTGTTGGTTCC | GTGAAATTGACACAGAAGCTGTTCATGTT | *Campbell et al. 2012* |
| OclWD_S15carpa1_437NC | WSCT | ACCATTTTCTGAAAGGTTCTTTT | AGACACCATTTTCTGAAAGGTTCTTTC | ATAATGCCACATCGGAAACATGGTT | *Campbell et al. 2012* |
| OclWD_Tnsf_387Kal | WSCT | AATTGTGGCAAAACTATGTTAATGC | ACAATTGTGGCAAAACTATGTTAATGT | GGTGACGTGTGAGTGGTTTGAACAT | *Kalinowski et al. 2011* |
| OclWD101119_Garza | WSCT | TGTCCTAGCTACTGCCACATGG | CTGTCCTAGCTACTGCCACATGA | ACTTTCATTTTCCACGTTTCTTTAGCCTA | *Campbell et al. 2012* |
| OclWD103713_Garza | WSCT | AGATGGAGAGCTCTCTGCCTTG | AGATGGAGAGCTCTCTGCCTTA | CCACAACAAACTCATTCATGCACTCCA | *Campbell et al. 2012* |
| OclWD111084_Garza | WSCT | CTAAGTTGTCGTATGTAGATATAAAACA | CCTAAGTTGTCGTATGTAGATATAAAACG | CCAGGGTGTTCTGGGTTTCTGGAT | *Pritchard et al. 2012; Campbell et al. 2012* |
| OclWD111312_Garza | WSCT | CATTACAGATGAGCTGGAGGG | CCATTACAGATGAGCTGGAGGC | AGTTCTGCCCTTGAACCTG | *Pritchard et al. 2012; Campbell et al. 2012* |
| OclWD120255_Garza | WSCT | GGAATTGAAGTTAGCTATCTATGACTATG | TGGAATTGAAGTTAGCTATCTATGACTATA | ACTCAAATGACTAAAACGTCAGTTACTGG | *Campbell et al. 2012* |
| OclWD96500L_Garza | WSCT | GGCGAGTGATTAAAAAGACAGACAG | GGCGAGTGATTAAAAAGACAGACAT | GACTCCAGAAAGCAGTAGAAGAAAATAAAT | *Pritchard et al. 2012; Campbell et al. 2012* |
| OmyWD_RAD_51821_Hoh | WSCT | AACTCCACAAGGTCAGAGGTAAC | AACTCCACAAGGTCAGAGGTAAA | CTACTCTGCCGACATCCTATCAGAA | *Hohenlohe et al. 2011; Amish et al. 2012* |
| OmyWD_RAD_52968_Hoh | WSCT | GGTGAATTCGGTGTTGTCTGC | CGGTGAATTCGGTGTTGTCTGT | GAGTCTCATCCCTGCAGGGCTT | *Hohenlohe et al. 2011; Amish et al. 2012* |
| FLU_OmyRD_RAD_17063_Hoh | WCT | GTCAGTAGGAGGTGCTATTGAGA | TCAGTAGGAGGTGCTATTGAGC | GCCTGCAGGCTGTCCAGTAGTT | *Hohenlohe et al. 2011; Amish et al. 2012* |
| FLU_OmyRD_RAD_17806_Hoh | RBT | TGGGCTGTGTGAGAGACAGAGA | TGGGCTGTGTGAGAGACAGAGT | ACACCTGCAGGGCCTGTCTGAA | *Hohenlohe et al. 2011; Amish et al. 2012* |
| FLU_OmyRD_RAD_19234_Hoh | RBT | TTCCTGTGTAAAGCAGTGGTGG | ACTTCCTGTGTAAAGCAGTGGTGA | GTGCTCGTACCATCCACCGTCAA | *Hohenlohe et al. 2011; Amish et al. 2012* |
| FLU_OmyRD_RAD_21431_Hoh | RBT | CTGTTCAGGGTGATGATGCTGT | CTGTTCAGGGTGATGATGCTGC | CAGGATGAGGGTTGCCTGGTCAT | *Hohenlohe et al. 2011; Amish et al. 2012* |
| FLU_OmyRD_RAD_23247_Hoh | RBT | CCCACGGGATACTGGGTG | CCCCACGGGATACTGGGTA | CCTGCAGGAGCTGGTCAGCTAT | *Hohenlohe et al. 2011; Amish et al. 2012* |
| FLU_OmyRD_RAD_26352_Hoh | RBT | ATGCACACCACTGCATCCAGAT | ATGCACACCACTGCATCCAGAC | CTACTGTTACAACCTGCAGGAGCTA | *Hohenlohe et al. 2011; Amish et al. 2012* |
| FLU_OmyRD_RAD_28080_Hoh | RBT | GATGTGTGGCTGTTGGTCAACCA | ATGTGTGGCTGTTGGTCAACCG | GCAAGACCCTCAGAATCCTCTTCAA | *Hohenlohe et al. 2011; Amish et al. 2012* |
| FLU_OmyRD_RAD_30378_Hoh | RBT | GGTCTGTCCCCCTGTCCGT | TCTGTCCCCCTGTCCGG | GCAGTGTGACCCTGCAGGACA | *Hohenlohe et al. 2011; Amish et al. 2012* |
| FLU_OmyRD_RAD_31988_Hoh | RBT | ATAATAAGATCATGCAACAGTAAGTGTTTG | ATAATAAGATCATGCAACAGTAAGTGTTTC | ATGCCCCTGCAGGCAAGCCATT | *Hohenlohe et al. 2011; Amish et al. 2012* |
| FLU_OmyRD_RAD_38362_Hoh | RBT | AACCCTCCATTCGTCACATTTAAC | CCAACCCTCCATTCGTCACATTTAAT | CTCTTCTATCTTGTTGACGTCGACCTT | *Hohenlohe et al. 2011; Amish et al. 2012* |
| FLU_OmyRD_RAD_39958_Hoh | RBT | TGGGTAATCACGAGGGTACATCT | GGTAATCACGAGGGTACATCG | CGTCCAGAGGAGCCAATGGCAT | *Hohenlohe et al. 2011; Amish et al. 2012* |
| FLU_OmyRD_RAD_46598_Hoh | RBT | AGGTCCATCAAGTCAAAGGCG | CCAGGTCCATCAAGTCAAAGGCA | GTGGATGACCACCTGCAGGACAA | *Hohenlohe et al. 2011; Amish et al. 2012* |
| FLU_OmyRD_RAD_57262_Hoh | RBT | CCACAGCGACCCCATCGAA | CCACAGCGACCCCATCGAG | GGTCAAATGTCAGGGTTAATCAGAAGTA | *Hohenlohe et al. 2011; Amish et al. 2012* |
| FLU_OmyRD_RAD_57673_Hoh | RBT | TCTTACCACGAGCTCAGGGAC | TCTTACCACGAGCTCAGGGAT | GCTGGATCTCATGGTGGTCCAGAT | *Hohenlohe et al. 2011; Amish et al. 2012* |
| FLU_OmyRD_RAD_60674_Hoh | RBT | TGTGCTGCAGCCCACATCAGAA | TGCTGCAGCCCACATCAGAG | TTAACCTGCAGGATGAGGAAGGCTT | *Hohenlohe et al. 2011; Amish et al. 2012* |
| FLU_OmyRD_RAD_69061_Hoh | RBT | GTCTGTAGCATATACTATGTTGTCCT | TCTGTAGCATATACTATGTTGTCCC | CCTGTCTGGGAATAACAGCCGTATA | *Hohenlohe et al. 2011; Amish et al. 2012* |
| FLU_OmyRD_RAD_77157_Hoh | RBT | TGTGTTACAGCTGCGGGTCCTT | TGTTACAGCTGCGGGTCCTG | GGTCAGGCTGCAGTGGAGGAAA | *Hohenlohe et al. 2011; Amish et al. 2012* |
| OclRD_Cal_155Kal | RBT | TTGTTTGATTTATTTTTATACAGTTGTATGCA | TGTTTGATTTATTTTTATACAGTTGTATGCT | TGATTGTAACRAGGGAATAAATTAAAAGGA | *Kalinowski et al. 2011* |
| OclRD_P53T7R2_Har | RBT | TGCACCAGTACGTTTTGGCCAATA | CACCAGTACGTTTTGGCCAATG | GTCAAATCAAAAACATGAAGAACGACCCAT | *Harwood and Phillips 2011* |
| OclRd_RAG1_233Kal | RBT | GGCTAATAATTGGGGCCTCAG | ATCTGGCTAATAATTGGGGCCTCAA | GGCCCACACAGCAGGCCATTTT | *Kalinowski et al. 2011* |
| OclRD_Thymo_320Kal | RBT | TTAATTAGCATTTTTGAATAGACTTAACCCA | TAATTAGCATTTTTGAATAGACTTAACCCG | GAAATGACGTGGAATCAACGTTGATTCAA | *Kalinowski et al. 2011* |
| OclRD_VIM_337Kal | RBT | CCGGCTCTCGTCGGATACG | CCCGGCTCTCGTCGGATACT | GCTTTGAACTCCGAGTTGATGGCAT | *Kalinowski et al. 2011* |
| OmyRD_F5_136May | RBT | ATCATTTGAACGAAAAATATAGACCTGATC | ATCATTTGAACGAAAAATATAGACCTGATG | CATTCATTAGCATTGCATTCTGGGGTTTT | *Campbell et al. 2012* |
| OmyRD_RAD_20663_Hoh | RBT | GGAGCAAAGCATTAAAAGTGTGCTG | ATGGAGCAAAGCATTAAAAGTGTGCTT | GTGCTCGTACCATCCACCGTCAA | *Hohenlohe et al. 2011; Amish et al. 2012* |
| OmyRD_RAD_22111_Hoh | RBT | GAACTTTGCTGGGCATGTGGG | GAACTTTGCTGGGCATGTGGT | TGCACGGATAACATGGTCTTTGATAACTT | *Hohenlohe et al. 2011; Amish et al. 2012* |
| OmyRD_RAD_29252_Hoh | RBT | GTCGTTCTTCTGGCCCAGGAC | TGTCGTTCTTCTGGCCCAGGAA | GTCAGGCTCTGACGGCCTACTT | *Hohenlohe et al. 2011; Amish et al. 2012* |
| OmyRD_RAD_42014_Hoh | RBT | GGTGAAAGTACAGGTAGCGCTTG | AGGTGAAAGTACAGGTAGCGCTTA | AACAGCTTACACCCAGAGCTGCTT | *Hohenlohe et al. 2011; Amish et al. 2012* |
| OmyRD_RAD_48301_Hoh | RBT | AGGGATGAGACTCCTCTGAAC | CAGGGATGAGACTCCTCTGAAG | TCTTCCTGCTGCTGATGTTGCTGTT | *Hohenlohe et al. 2011; Amish et al. 2012* |
| OmyRD_RAD_49759_Hoh | RBT | GTCTTTGTTGGAATTTATTGCCATATTC | TCTTTGTTGGAATTTATTGCCATATTA | ATATCTCACCTGCAGGTTTAAGTACCAAA | *Hohenlohe et al. 2011; Amish et al. 2012* |
| OmyRD_RAD_51740_Hoh | RBT | TATCGGGTACCTGCAGGTGAC | TATCGGGTACCTGCAGGTGAG | GCCTTGACAGTACAACAGGCACTTT | *Hohenlohe et al. 2011; Amish et al. 2012* |
| OmyRD_RAD_54584_Hoh | RBT | GTACCTGCAGGGAAAGCTACTCT | TACCTGCAGGGAAAGCTACTCG | GGATCCACCAGTGTGTATGTGTAGTT | *Hohenlohe et al. 2011; Amish et al. 2012* |
| OmyRD_RAD_55820_Hoh | RBT | GAGGCCTTACAGATTGATTGCACA | AGGCCTTACAGATTGATTGCACG | GGCACAGCAGAAGACCAATTTCCAT | *Hohenlohe et al. 2011; Amish et al. 2012* |
| OmyRD_RAD_5666_Hoh | RBT | GGAGGAACCTGCAGGTGGC | GAGGAACCTGCAGGTGGG | AAAGTCAGTTAACTACACTACAGACCAATT | *Hohenlohe et al. 2011; Amish et al. 2012* |
| OmyRD_RAD_59515_Hoh | RBT | AGGTGGTGCCAGGACAGGG | CAAGGTGGTGCCAGGACAGGT | CCAGATCCAGGCCTGCAGGTAA | *Hohenlohe et al. 2011; Amish et al. 2012* |
| OclYD_CLK3Y1_Har | YSCT | GCTGGGACTGCTGCTGGG | GCTGGGACTGCTGCTGGT | TCTGCAGGGCAGCACTCCACA | *Harwood and Phillips 2011* |
| OclYGD100974_Garza | YSCT | TAG1-GTTTAAGTATTGTGATACATGCAAATTAAGTG | TAG2-TGTTTAAGTATTGTGATACATGCAAATTAAGTA | CTGTGAACTCTATTTCATAGCATAACAT | *Pritchard et al. 2012; Campbell et al. 2012* |
| OclYGD104216_Garza | YSCT | GAGCTGTAGCAGCAGGATCAGTA | AGCTGTAGCAGCAGGATCAGTG | AGGTCATTGTGTACGGAGGAGTGAT | *Pritchard et al. 2012; Campbell et al. 2012* |
| OclYGD104569_Garza | YSCT | AACGTGAACAACATGCAGGACAG | ACAACGTGAACAACATGCAGGACAA | ATAATCYTCCAGAACCACCTCCACTTT | *Campbell et al. 2012* |
| OclYGD106419_Garza | YSCT | AACCCTGGTTGTAATGCATGTAATA | CAACCCTGGTTGTAATGCATGTAATG | AATCACACGGCTGACGGACACTT | *Pritchard et al. 2012; Campbell et al. 2012* |
| OclYGD109525_Garza | YSCT | GATGGAGAGGTGTACCCACCG | ATGGAGAGGTGTACCCACCT | GAGGGTAGTGCATATCAGCCCCAA | *Campbell et al. 2012* |
| OclYGD110571_Garza | YSCT | AAAAAACAGCAAACAAAGCATATTAAATTC | CAAAAAACAGCAAACAAAGCATATTAAATTA | TAATGGACTCTTAACAACCCCAGATTGTA | *Pritchard et al. 2012; Campbell et al. 2012* |
| OclYGD112820_Garza | YSCT | CTTAGTCTCTGGGGAAATGAAC | ACCTTAGTCTCTGGGGAAATGAAT | ACTAATAACAATTTGACCGCGCCGC | *Campbell et al. 2012* |
| OclYGD113600_Garza | YSCT | TCTCTCAGATTGAAAGACTACAAAAAACAA | CTCTCAGATTGAAAGACTACAAAAAACAG | CAGGCATGTTTGTAGGTTCATAGTCTTAA | *Campbell et al. 2012* |
| OclYGD117286_Garza | YSCT | TGGCATTATTCTGACTTGAGATCTATG | TTTTGGCATTATTCTGACTTGAGATCTATT | CTCGATCTCAATGGTAGACTTCTTTAGTT | *Campbell et al. 2012* |
| OclYGD117370_Garza | YSCT | GCTTGTTCTTGGGGGTAGGAG | GCTTGTTCTTGGGGGTAGGAA | CACCGTGCCCCTGTCCCACAT | *Pritchard et al. 2012; Campbell et al. 2012* |
| OclYGD129458_Garza | YSCT | CATTTATCCCCTTTTTTTGTTTGTTTAG | CCATTTATCCCCTTTTTTTGTTTGTTTAC | CCCACAAAAAGCAACTGGACATCT | *Pritchard et al. 2012; Campbell et al. 2012* |
| OclYGD131785_Garza | YSCT | GATTGTTGCACATCCCTTAATGTTTGT | ATTGTTGCACATCCCTTAATGTTTGA | GCTGGTTTCAGTTTGAGGGCTGATA | *Pritchard et al. 2012; Campbell et al. 2012* |
| OclYSD105385_Garza | YSCT | CGGGACTTGGATACGACCG | TTCCGGGACTTGGATACGACCA | CACAGTTTGCTCTGAGCAGTCTGTA | *Campbell et al. 2012* |
| OclYSD107607_Garza | YSCT | ATTTTCCGGAAACCTCTCATCACTT | ATTTTCCGGAAACCTCTCATCACTG | CCTACATCGCTGGAGAACATGGAA | *Pritchard et al. 2012; Campbell et al. 2012* |
| OclYSD116865_Garza | YSCT | AACAGGCAACATTGAAATGAAGACT | CAACAGGCAACATTGAAATGAAGACG | TAGTCCTGTTGTCATAACTATTT | *Campbell et al. 2012* |
| OclYSD117432_Garza | YSCT | AGTTCACCATGAGGAATAAAGGTGC | AGTTCACCATGAGGAATAAAGGTGT | CGTCCAGGCTGTGGTACTGGTA | *Pritchard et al. 2012; Campbell et al. 2012* |
| OclYSD129870_Garza | YSCT | GGACCATATCAAMTGAGTATCATATTATTAAT | ACCATATCAAMTGAGTATCATATTATTAAC | AGATACTGTACACTGTATTAGCCTCAGTT | *Pritchard et al. 2012; Campbell et al. 2012* |
